# Anti-CDCP1 immuno-conjugates for detection and inhibition of ovarian cancer

**DOI:** 10.7150/thno.30736

**Published:** 2020-01-12

**Authors:** Brittney S. Harrington, Yaowu He, Tashbib Khan, Simon Puttick, Paul J. Conroy, Thomas Kryza, Tahleesa Cuda, Kamil A Sokolowski, Brian WC Tse, Katherine K. Robbins, Buddhika J. Arachchige, Samantha J. Stehbens, Pamela M. Pollock, Sarah Reed, S. John Weroha, Paul Haluska, Carlos Salomon, Rohan Lourie, Lewis C. Perrin, Ruby H. P. Law, James C. Whisstock, John D Hooper

**Affiliations:** 1 Mater Research Institute - The University of Queensland, Translational Research Institute, Woolloongabba, QLD 4102, Australia.; 2 Mater Ovarian Cancer Research Collaborative, Mater Adult Hospital, South Brisbane, QLD 4101, Australia.; 3 Australian Institute for Bioengineering and Nanotechnology, The University of Queensland, St Lucia, Australia.; 4 Commonwealth Scientific and Industrial Research Organisation, Probing Biosystems Future Science Platform, Herston, QLD 4029, Australia.; 5 Biomedicine Discovery Institute and Department of Biochemistry and Molecular Biology, Monash University, Melbourne, VIC 3800, Australia.; 6 Preclinical Imaging Facility, Translational Research Institute, Woolloongabba, QLD 4102, Australia.; 7 Centre for Clinical Research, University of Queensland, Herston, Qld 4029, Australia.; 8 Institute of Health and Biomedical Innovation, Queensland University of Technology, Woolloongabba, QLD 4102, Australia.; 9 Department of Medical Oncology, Mayo Clinic, Rochester, MN, USA.; 10 Merck Research Laboratories, Rahway, NJ, USA.; 11 Mater Health Services, South Brisbane, QLD 4101, Australia.

**Keywords:** CDCP1, ovarian cancer, immuno-conjugate, antibody

## Abstract

CUB-domain containing protein 1 (CDCP1) is a cancer associated cell surface protein that amplifies pro-tumorigenic signalling by other receptors including EGFR and HER2. Its potential as a cancer target is supported by studies showing that anti-CDCP1 antibodies inhibit cell migration and survival *in vitro*, and tumor growth and metastasis *in vivo*. Here we characterize two anti-CDCP1 antibodies, focusing on immuno-conjugates of one of these as a tool to detect and inhibit ovarian cancer.

**Methods**: A panel of ovarian cancer cell lines was examined for cell surface expression of CDCP1 and loss of expression induced by anti-CDCP1 antibodies 10D7 and 41-2 using flow cytometry and Western blot analysis. Surface plasmon resonance analysis and examination of truncation mutants was used to analyse the binding properties of the antibodies for CDCP1. Live-cell spinning-disk confocal microscopy of GFP-tagged CDCP1 was used to track internalization and intracellular trafficking of CDCP1/antibody complexes. *In vivo*, zirconium 89-labelled 10D7 was detected by positron-emission tomography imaging, of an ovarian cancer patient-derived xenograft grown intraperitoneally in mice. The efficacy of cytotoxin-conjugated 10D7 was examined against ovarian cancer cells *in vitro* and *in vivo*.

**Results**: Our data indicate that each antibody binds with high affinity to the extracellular domain of CDCP1 causing rapid internalization of the receptor/antibody complex and degradation of CDCP1 via processes mediated by the kinase Src. Highlighting the potential clinical utility of CDCP1, positron-emission tomography imaging, using zirconium 89-labelled 10D7, was able to detect subcutaneous and intraperitoneal xenograft ovarian cancers in mice, including small (diameter <3 mm) tumor deposits of an ovarian cancer patient-derived xenograft grown intraperitoneally in mice. Furthermore, cytotoxin-conjugated 10D7 was effective at inhibiting growth of CDCP1-expressing ovarian cancer cells *in vitro* and *in vivo*.

**Conclusions**: These data demonstrate that CDCP1 internalizing antibodies have potential for killing and detection of CDCP1 expressing ovarian cancer cells.

## Introduction

Elevated expression of the cellular receptor CUB-domain containing protein 1 (CDCP1) is associated with poor prognosis in epithelial cancers of the lung 1, 2, kidney 3, pancreas 4, 5, breast 6, 7 and ovary 8-11. Based on data from *in vitro* and animal models, CDCP1 is functionally important for each of these cancers by promoting cell survival and metastasis 11-14, as well as resistance to chemotherapy 11-15 and targeted agents 16-19. The elevated expression of CDCP1 on the surface of malignant cells has led to its investigation in pre-clinical models as a target for monoclonal antibodies (mAbs) that have therapeutic potential against prostate, breast and epithelial ovarian cancer (EOC) 9-12, 15, 20-24.

The structural features of CDCP1 support its potential as a target for antibody based anti-cancer agents. It is predominantly located on the cell surface and after removal of its 29 residue signal peptide, CDCP1 spans 807 residues including a 637 residue amino-terminal extracellular domain (ECD), a 20 residue transmembrane domain, and a 150 residue carboxyl-terminal intracellular domain 25, 26. The intracellular region of CDCP1 is critical for its interactions with a range of key signalling proteins. These include the kinase Src which is a key regulator of CDCP1-mediated signalling in pathological settings including cancer. CDCP1 is phosphorylated by Src at tyrosine 734 (Y734) and then Y743 and Y762 27. These phosphorylation events occur in response to a range of cellular processes that promote cancer progression including reduced cell adhesion during mitosis and cell shedding 28, cell de-adhesion 14, 29, 30, cleavage of 135 kDa CDCP1 to generate a 75 kDa cell retained fragment 12, 31, and oncogenic transformation 21. Src phosphorylation of CDCP1 is followed rapidly by docking of PKCδ to the intracellular domain of CDCP1. Highlighting the importance of these events, formation of the CDCP1/Src/PKCδ complex is accompanied by further cancer promoting signal transduction including via the kinase FAK during loss of cell adhesion 32, the cell-matrix adhesion protein β1 integrin during vascular metastasis 13, the receptor tyrosine kinase HER2 in therapy resistant breast cancer 16 and the kinase Akt in cancer cell survival 11, 12, 26, 33.

CDCP1 is a potential target in EOC for therapeutic mAbs as it is expressed on the cell surface of the malignant component of the vast majority of these tumors and is not expressed by normal ovary and fallopian tube 8-11. Also, it is functionally important in this malignancy, promoting EOC cell migration, survival, spheroid formation and chemotherapy resistance *in vitro*, and tumor growth and metastasis *in vivo* 8-11. Recently, we have shown that the anti-CDCP1 mAb 10D7 reduces tumor burden in cell line and patient derived xenograft mouse models of EOC 10, 11. Our aim in this study was to examine the suitability of CDCP1 as a theranostic target for imaging and treatment of EOC. To achieve this aim we have characterized 10D7, and a second anti-CDCP1 mAb, 41-2, for the ability to bind to CDCP1 and induce its internalization and degradation. We have also defined the ability of 10D7 to be used for targeted cytotoxin delivery to EOC cells *in vitro* and* in vivo*, and for PET-CT imaging of mouse models of EOC.

## Results

### 41-2 and 10D7 cause loss of cell surface CDCP1

**Figure 1:**
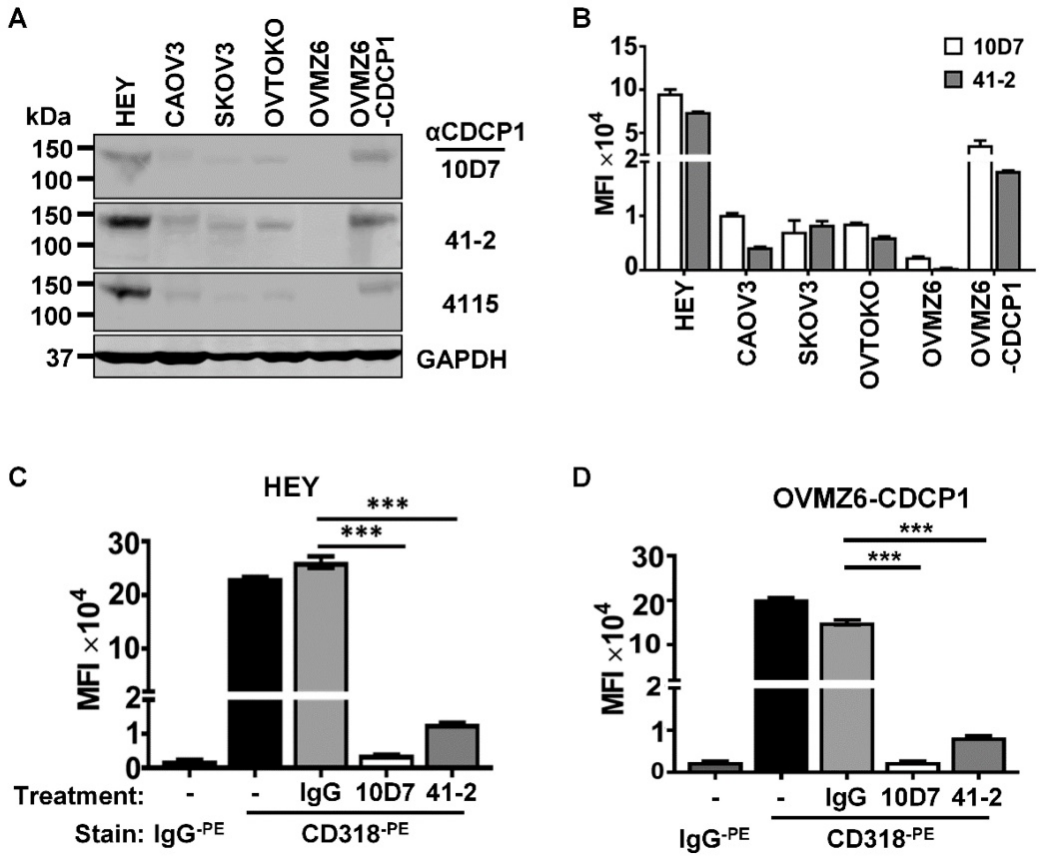
** Antibody-induced loss of CDCP1 from the cell surface.** (**A**) Western blot analysis of lysates from the indicated cell lines using two mouse monoclonal anti-CDCP1 antibodies, 10D7 and 41-2 (1 µg/ml), a commercial rabbit polyclonal anti-CDCP1 antibody, 4115 (1:2,000 dilution), and an anti-GAPDH antibody (1:10,000). (**B**) Flow cytometry analysis of the indicated cell lines for plasma membrane localized CDCP1 using 10D7 and 41-2. Fixed cells were stained with the respective anti-CDCP1 antibody followed by an APC-conjugated anti-mouse IgG, then analysed by flow cytometry. Data are displayed graphically as MFI values corrected for background signal determined from cells stained with only the APC-conjugated anti-mouse IgG. 10D7 and 41-2 identified the same proportion of CDCP1 expressing cells as: HEY 89%; CAOV3 93%; SKOV3 99%; OVTOKO 86%; OVZM6 0%; OVMZ6-CDCP1 75%. (**C, D**) Flow cytometry analysis of HEY (c) and OVMZ6-CDCP1 (d) cells treated for 30 minutes at 37°C with 10D7, 41-2 or control IgG_1_κ (5µg/ml). Treated cells were fixed and plasma membrane localized CDCP1 detected using fluorescently tagged anti-CDCP1 antibody CD318^-PE^. Background signal was assessed by staining treated cells with fluorescently tagged control IgG (IgG^-PE^). Data are displayed as MFI values. All data are mean ± SEM from three independent experiments. ***P<0.001.

Western blot and flow cytometry analyses were employed to select EOC cell lines to examine the mechanism of action of anti-CDCP1 mAbs 41-2 and 10D7. Assays were performed on the five EOC cell lines HEY, CAOV3, SKOV3, OVTOKO and OVMZ6, as well as OVMZ6 cells stably expressing CDCP1 (designated OVMZ6-CDCP1). As shown in Fig. 1A, total CDCP1 was detected at similar relative levels by these antibodies, with HEY cells expressing CDCP1 at highest levels, and OVMZ6 cells the only non-expressing line. Another antibody, 4115 from a commercial supplier, that recognises the intracellular carboxyl terminal of CDCP1, also detected CDCP1 at approximately the same relative level as 41-2 and 10D7 (Figure 1A). Flow cytometry analysis indicated that cell surface CDCP1 levels were consistent with total levels detected by Western blot analysis. As shown in Figure 1B, cell surface levels of CDCP1 detected with 10D7 and 41-2, were up to ~10-fold higher in HEY cells compared with CAOV3, SKOV3 and OVTOKO cells. OVMZ6-CDCP1 cells displayed cell surface CDCP1 levels about half those of endogenous expressing HEY cells (Figure 1B).

Based on these analyses the highest expressing lines, HEY and OVMZ6-CDCP1, were selected to examine the impact of 41-2 and 10D7 on cell surface CDCP1. This was performed on adherent cells at 37°C treated with 10D7, 41-2 or isotype control immunoglobulin (IgG)_1_κ (5µg/ml) for 30 minutes. As shown in Figure 1C and D, flow cytometry analysis, performed on chilled cells to prevent further loss of cell surface CDCP1, using antibody CD318, tagged with the fluorophore phycoerythrin (CD318^-PE^) and generated against the CDCP1 ECD, indicated that 10D7 caused almost complete loss of cell surface CDCP1, while 41-2 reduced cell surface levels by ~90%. In contrast, IgG_1_κ had no effect on CDCP1.

### 41-2 and 10D7 induce internalization and degradation of CDCP1

**Figure 2:**
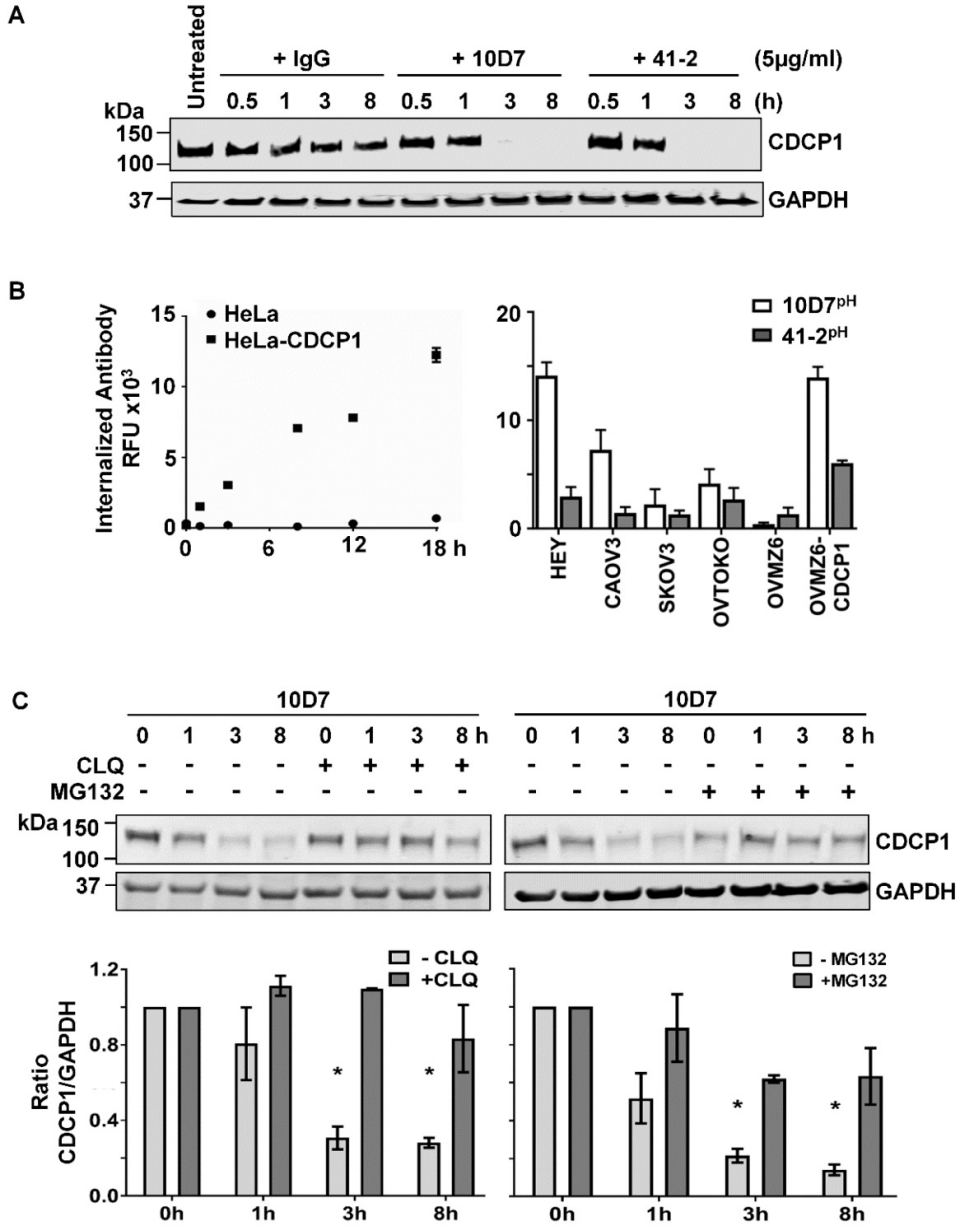
** Degradation of CDCP1 induced by internalizing mAbs 41-2 and 10D7.** (**A**) Western blot analysis, using anti-CDCP1 antibody 4115 (1:2,000) and an anti-GAPDH antibody (1:10,000), of lysates from HEY cells treated with isotype matched control IgG (*lef*t), 10D7 *(middle)* or 41-2 *(right)* for the indicated times. (**B**) Graph of fluorescence versus time from HeLa and HeLa-CDCP1 cells treated with 10D7^pH^ (5µg/ml) (*left*), and graph of fluorescence signal from six EOC cell lines following treatment with 10D7^pH^ or 41-2^pH^ (5µg/ml) for 8 hours (*right*). RFU, relative fluorescence units. (**C**) Impact of lysosomal (*left*) and proteasomal (*right*) inhibition on antibody-induced degradation of CDCP1. *Top panel*, Anti-CDCP1 (1:2,000) and -GAPDH (1:10,000) Western blot analysis of HEY cells treated with 10D7 in the presence or absence of the lysosomal inhibitor chloroquine (CLQ; 50 µM), or the proteasomal inhibitor MG132 (20 µM) for the indicated times. *Bottom panel*, Graph of the ratio of CDCP1 to GAPDH signal generated from Western blot analyses of lysates from three independent assays assessing the effect of CLQ and MG132 on 10D7-induced degradation of CDCP1. All data represent mean ± SEM from three independent experiments. *P<0.05.

We next examined the impact of 10D7 and 41-2 on total CDCP1 levels and whether mAb-induced loss of cell surface CDCP1 is accompanied by its degradation. This was assessed by Western blot analysis of lysates from HEY cells treated with antibody for 30 min to 8 h. As shown in Figure 2A, both antibodies caused complete loss of CDCP1 within the 3-8 h time period. In contrast, control IgG had no impact on CDCP1 levels (Figure 2A). To examine the mechanism of mAb-induced loss of CDCP1, we employed a fluorescence-based internalization assay using mAbs labelled with a pH-sensitive dye, pHAb, which at neutral pH is not fluorescent. However, its fluorescence increases significantly as acidity increases during protein trafficking into endosomes (pH 6.0-6.5) and lysosomes (pH 4.5-5.5) 34, 35. We first assessed the impact of the dye on antigen recognition by comparing fluorescence obtained from non-CDCP1 expressing cells (HeLa) versus this line engineered to stably express CDCP1 (HeLa-CDCP1). Incubation of these cells with 10D7^pH^ resulted in increasing signal over an 18 h period from HeLa-CDCP1 but not parental HeLa cells, indicating that the pH-labelled mAb is able to recognize cell surface CDCP1 and it becomes internalized into low pH vesicles (Figure 2B left). The assay was then performed on the six EOC cell lines using both 10D7^pH^ and 41-2^pH^. As shown in Figure 2B (right), after 8 h both antibodies induced robust fluorescence, indicating internalization to low pH vesicles. The signal was generally in proportion to the level of cell surface CDCP1 observed in Figure 1B. Of note, for each of the five CDCP1 expressing lines, mAb trafficking to endosomes/lysosomes was greatest for 10D7^pH^ (Figure 2C, right), suggesting this mAb is more effective than 41-2 at undergoing internalization.

We have shown that under basal conditions, endogenous CDCP1 is constitutively internalized then recycled to the plasma membrane or degraded via the proteasome 8. In contrast, our fluorescence-based analyses (Figure 2B), indicated that 10D7 and 41-2 internalize to endosomes/lysosomes. Thus, we were interested in the relative contribution of lysosomal and proteasomal mechanisms to mAb-induced CDCP1 degradation. HEY cells were treated with 10D7 combined with chloroquine (CLQ) to inhibit lysosomal degradation, or MG132 to inhibit proteasomal degradation. We selected 10D7 for this assay because of its greater efficiency at trafficking to endosomes/lysosomes compared with 41-2. As shown in Figure 2C, both lysosomal and proteasomal inhibition stabilized CDCP1 in response to 10D7-induced degradation, with lysosomal inhibition slightly more efficient.

### 10D7 and 41-2 bind with high affinity within amino acids 30 to 358 of CDCP1

**Figure 3:**
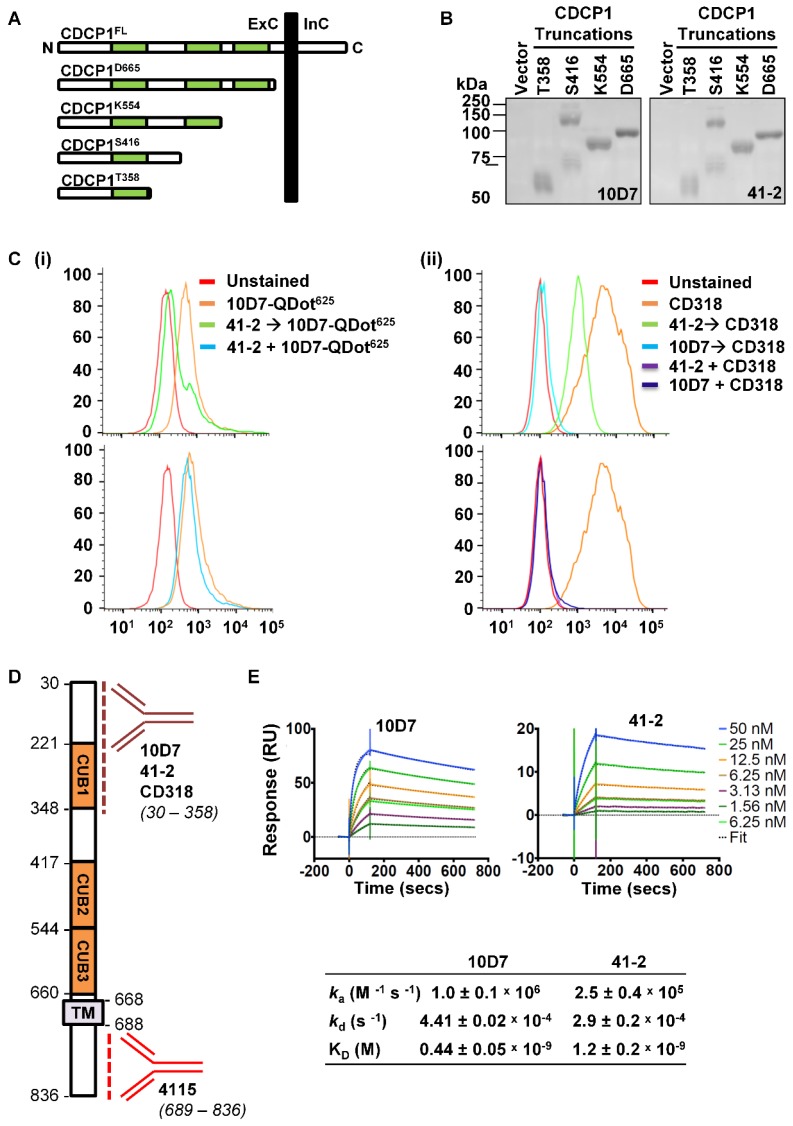
** 10D7 and 41-2 bind with high affinity to the ECD of CDCP1.** (**A**) Schematic representation of full length CDCP1 (CDCP1^FL^) and progressively shorter carboxyl terminal truncations (CDCP1-T358, -S416, -K554, -D665). CUB domains are colored green. (**B**) 10D7 and 41-2 (1 µg/ml) Western blot analysis of conditioned media from OVMZ6 cells transiently transfected with a control vector of constructs encoding CDCP1-T358, -S416, -K554, or -D665. (**C**) Flow cytometry analysis of HEY cells incubated with: (i) 10D7-QDot^625^ for 1 h, unlabelled 41-2 for 1 h then 10D7-QDot^625^ for 1 h, or concurrently with 10D7-QDot^625^ and 41-2 for 1 h; or (ii) CD318^-PE^ for 1 h, unlabelled 10D7 or 41-2 for 1 h then CD318^-PE^ for 1 h, or concurrently with 10D7 or 41-2 and CD318^-PE^ for 1 h. (**D**) Schematic of CDCP1 showing the regions to which antibodies 10D7, 41-2, CD318 and 4115 bind. (**E**) *Top panels*, Sensorgrams of CDCP1-ECD (concentration range 1.56 to 50 nM) binding to immobilized 10D7 *(left)* and 41-2 *(right)* depicting association (increasing signal) and dissociation (reducing signal) over time. *Bottom panel*, Table of kinetic parameters. k_a_, association rate; k_d_, dissociation rate; K_D_, affinity constant.

To provide additional mode of action information, we next examined the 10D7 and 41-2 binding sites and affinities. Binding sites were examined by Western blot analyses of conditioned media from OVMZ6 cells transiently expressing progressively shorter carboxyl terminal truncations of CDCP1 including: the complete CDCP1 ECD (CDCP1-D665); two of the three CUB-like domains (CDCP1-K554); and one of these domains (CDCP1-S416 and -T358) (Figure 3A). As shown in Figure 3B, both 41-2 and 10D7 detected each of the CDCP1 truncations, with the CDCP1-S416 truncation apparent as a monomer of ~70 kDa, and dimer of ~140 kDa. As the CDCP1 signal peptide, spanning residues 1 to 29, is removed during cellular processing, these data indicate that both antibodies bind within amino acid 30 to 358 of CDCP1 within its ECD.

We next employed two flow cytometry assay formats, using high CDCP1 expressing HEY cells, to examine whether 10D7 and 41-2 compete for binding sites on CDCP1, and whether either competes with antibody CD318^-PE^ for binding to CDCP1. The first assay involved incubating cells with the unlabelled competing antibody followed by fluorescently labelled 10D7 (10D7-QDot^625^) or CD318^-PE^ as the detecting antibody. In the second format, cells were co-incubated with the unlabelled competing antibody and the fluorescently labelled detecting antibody. All antibodies were used at saturating concentrations in both assay formats. In assays in which cells were incubated with 41-2 followed by 10D7-QDot^625^ there was a shift in MFI to almost background levels (Figure 3C(i) top), indicating that pre-bound 41-2 is able to largely block 10D7 binding to CDCP1. In contrast, the MFI value was largely unaffected when cells were co-incubated with 41-2 and 10D7-QDot^625^ (Figure 3C(i) bottom), indicating that 10D7 associates more rapidly with CDCP1 than 41-2. The data suggest that 41-2 and 10D7 compete for the same epitopes and that 10D7 likely associates more rapidly with CDCP1 and has higher affinity for this receptor. Interestingly, when cells were pre-incubated with 10D7 before incubation with CD318^-PE^, there was complete reduction in the MFI value to background levels, whereas there was only a partial reduction when cells were pre-incubated with 41-2 (Figure 3C(ii) top). This suggests that CD318^-PE^ can partially displace 41-2 but not 10D7. Also of note, co-incubation of CD318^-PE^ with 10D7 or 41-2 resulted in a complete reduction in the MFI value to background levels (Figure 3C(ii) bottom). As summarized in Fig 3D, collectively these results indicate that 10D7 and 41-2, which bind within amino acid 30 to 358, compete with CD318^-PE^ for binding sites on CDCP1. While 10D7 and 41-2 associate more rapidly than CD318^-PE^ to CDCP1, CD318^-PE^ can partially displace 41-2 but not 10D7, which suggests that 10D7 has higher affinity for CDCP1 than CD318.

To determine 41-2 and 10D7 affinity (K_D_) surface plasmon resonance analysis was performed using the immobilized mAbs and serial dilutions of the CDCP1-ECD (50 to 1.56 nM) as the analyte. As shown in Figure 3E, both antibodies had fast association (*k*_a_) and slow dissociation (*k*_d_) rates, with 10D7 displaying 2.7-fold higher affinity for CDCP1 than 41-2 with the respective K_D_ values 0.44 nM and 1.2 nM. The stronger affinity and slightly faster association and slower dissociation rates of 10D7, compared with 41-2, explain the ability of the former to outcompete 41-1 for binding to CDCP1 that is apparent in the flow cytometry data in Figure 3C(i) bottom, and the almost complete inability of 10D7 to displace pre-bound 41-2 noted in Figure 3C(i) top.

### 10D7 induces rapid clustering and lysosomal trafficking of CDCP1

**Figure 4:**
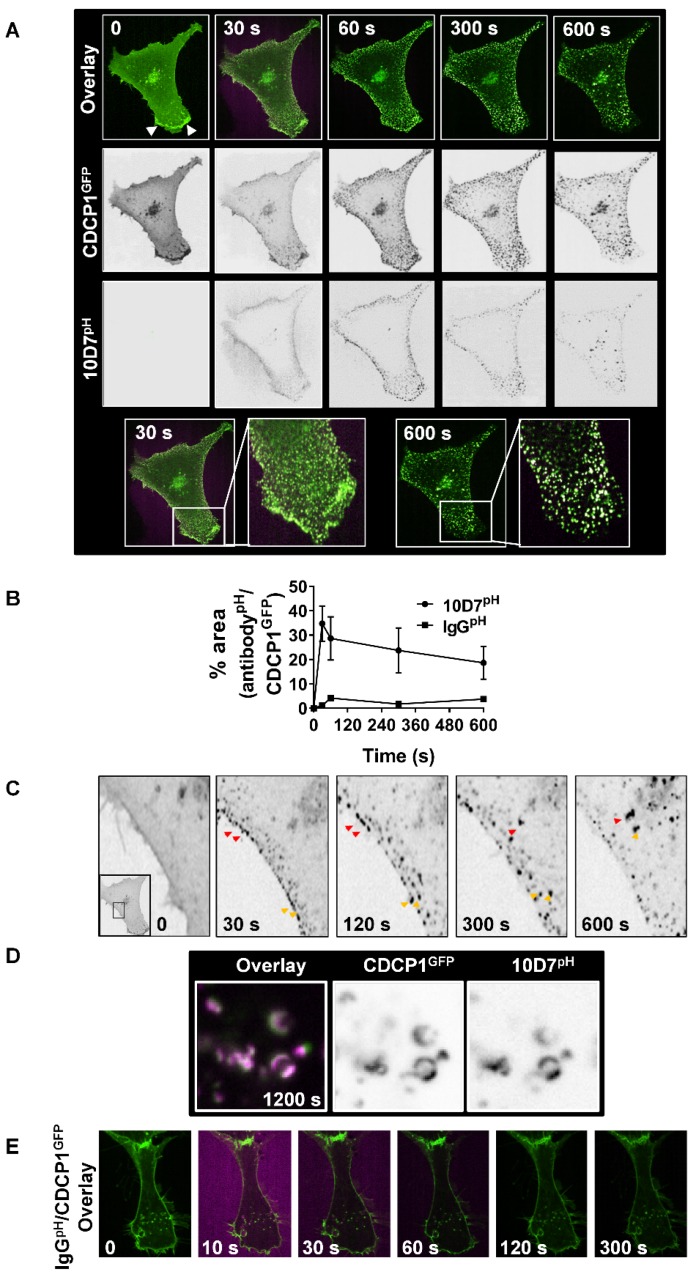
** 10D7-induces cell surface rapid clustering and lysosomal trafficking of CDCP1.** (**A**) Live-cell confocal microscopy images of HEY-CDCP1^GFP^ cells treated with 10D7^pH^ (5µg/ ml). Internalization of CDCP1^GFP^ and 10D7^pH^ was observed at 1 frame per second for 600 s. Insets highlight green punctate CDCP1^GFP^ positive cellular structures at 30 s, and white cellular structures at 600s that are positive for both CDCP1^GFP^ and 10D7^pH^. (**B**) Graph of complex formation between IgG^pH^ and CDCP1^GFP^ determined as the percentage of IgG^pH^ signal coincident with CDCP1^GFP^ signal using ImageJ software analysis. (**C**) Images of the plasma membrane and proximal cytoplasmic region of HEY-CDCP1^GFP^ cells indicating 10D7-induced clustering of CDCP1. In untreated cells CDCP1^GFP^ is located diffusely on the cell surface. In treated cells, arrowheads highlight rapid 10D7-induced clustering of CDCP1^GFP^ and its internalization. (**D**) *Left panel*, Overlay of CDCP1^GFP^ (green) and 10D7^pH^ (magenta) signals in HEY-CDCP1^GFP^ cells after 20 minutes of treatment showing co-localization of within endosomal-like structures. *Middle panel*, Black and white image of CDCP1^GFP^ signal. *Right panel*, Black and white image of 10D7^pH^ signal. (**E**) Live-cell confocal microscopy images of HEY-CDCP1^GFP^ cells treated with IgG7^pH^ (5µg/ ml). No internalization of CDCP1^GFP^ was observed within 300 s of treatment with IgG7^pH^.

Subsequent assays focus on 10D7-induced effects because of its greater ability, compared with 41-2, to mediate loss of CDCP1 from the cell surface (Figure 1C and D), traffic to low pH vesicles (Figure 2B), and higher affinity for CDCP1 (Figure 3D). To track internalization and degradation of mAb/CDCP1 complexes, we performed live-cell spinning-disk confocal microscopy of cells stably expressing CDCP1 tagged at the carboxyl terminal with GFP (CDCP1^GFP^). These assays were performed in HEY cells because we already understood key aspects of antibody-induced degradation of endogenous CDCP1 in this line including the rate of degradation and the contribution of lysosomal and proteasomal mechanisms to these processes. Untreated HEY-CDCP1^GFP^ cells displayed generally diffuse plasma membrane localization of CDCP1^GFP^ (green) with prominent accentuation of signal at regions of membrane ruffling (Figure 4A; time 0, white arrowheads, overlay and CDCP1^GFP^ panels). Within 30 seconds of 10D7^pH^ treatment (5 µg/ml), prominent clustering of CDCP1^GFP^ was apparent (Figure 4A, 30 s; and inset). Concomitantly, there was almost complete loss of CDCP1^GFP^ at membrane ruffles. In these images coincident signal of CDCP1^GFP^ (green) and 10D7^pH^ (magenta) is apparent as white puncta, indicating formation of 10D7/CDCP1 complexes. After 300 seconds, 10D7^pH^ fluorescence was further increased indicating its increasing internalization and accumulation in acidified intracellular vesicles (Figure 4A, 300 s, 10D7^pH^ panels). Over time, the size of white puncta increased indicating increased accumulation of CDCP1^GFP^/10D7^pH^ complexes within low pH intracellular vesicles (Figure 4A, 600 s, inset panel). Quantitative analysis indicated that formation of CDCP1^GFP^/10D7^pH^ complexes occurred rapidly with maximum overlap after approximately 30 seconds and this was sustained to 600 seconds (Figure 4B). High-resolution microscopy analysis further highlighted the 10D7-induced clustering of CDCP1^GFP^ occurring rapidly at the plasma membrane is followed by trafficking of 10D7/CDCP1 complexes into the cytoplasm (Figure 4C, red and yellow arrows). It also delineated trafficking of 10D7/CDCP1 complexes to intracellular vesicles, with signal from CDCP1^GFP^ (green) and 10D7^pH^ (magenta) largely coincident and localised to vesicle membranes (Figure 4D). In contrast with this rapid 10D7-induced trafficking, IgG^pH^ had no impact on CDCP1 location (Figure 4E).

### Tyrosine phosphorylation likely via Src is involved in 10D7-induced internalization and degradation of CDCP1

**Figure 5:**
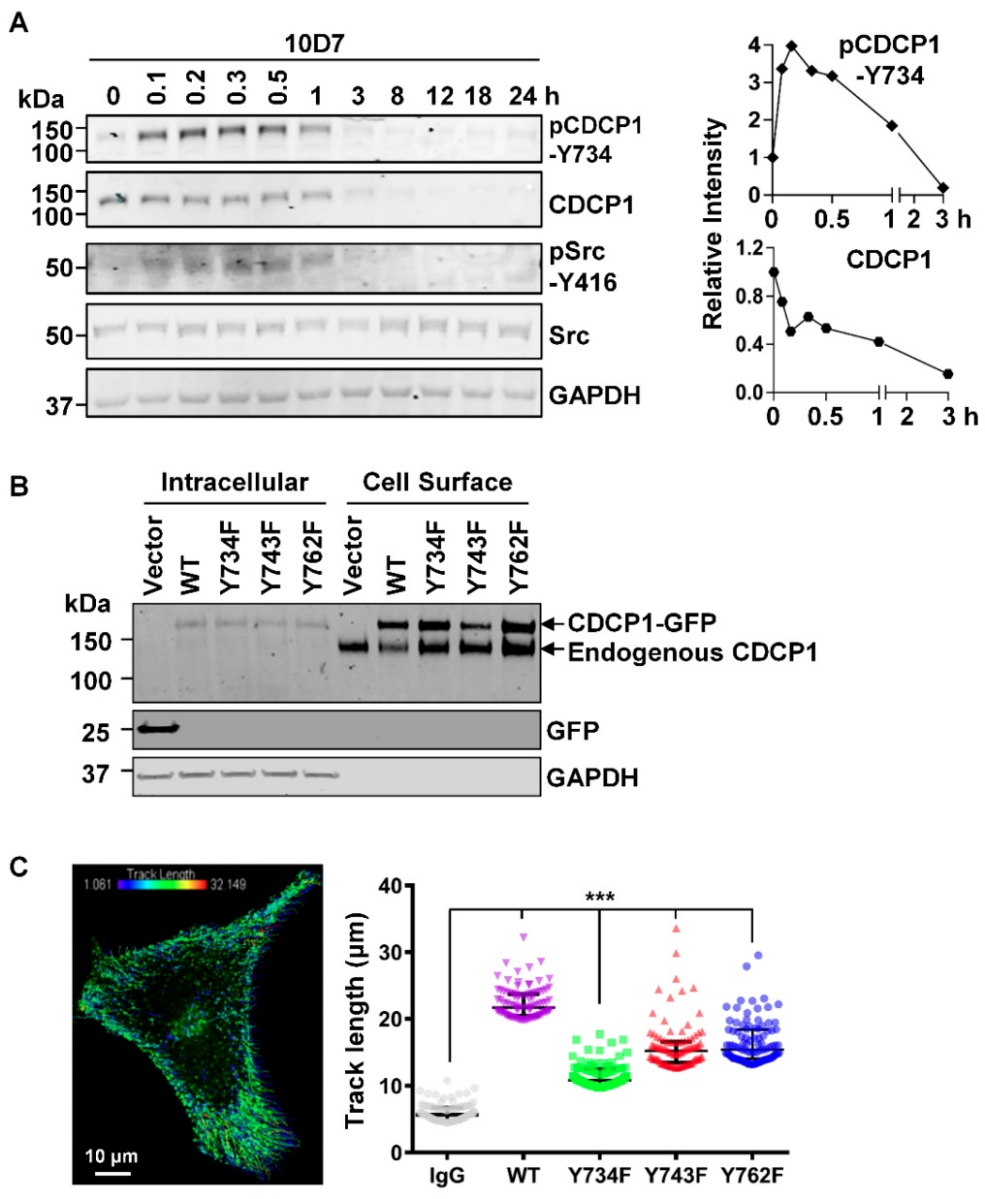
** CDCP1 is tyrosine phosphorylated during 10D7-induced internalization and degradation.** (**A**) Lysates from HEY cells treated with 10D7 (5µg/ml) for the indicated times were examined by Western blot analysis for CDCP1, p-CDCP1-Y734, Src, p-Src-Y416, and GAPDH. Antibody dilution was 1:2,000 except the anti-GAPDH antibody which was 1:10,000. The graphs display CDCP1 and p-CDCP1-Y734 levels determined by densitometric analysis with data representing mean ± SEM from three independent experiments. (**B**) Anti-CDCP1 (1:2,000), -GFP (1:2,000) and -GAPDH (1:10,000) Western blot analysis of fractions collected by cell surface biotinylation of HEY cells expressing CDCP1^GFP^, CDCP1^GFP^-Y734F, -Y743F or -Y762F. (**C**) Analysis of semi-automated computer tracking of CDCP1^GFP^ and CDCP1^GFP^-Y734F, -Y743F and -Y762F in response to 10D7. *Left*, representative image of CDCP1^GFP^ tracks that internalized in response to 10D7^pH^ in HEY cells. The image is an overlay onto cells of color-coded tracks (violet, tracks that moved the shortest distance; red, the tracks that moved the greatest distance). *Right*, Graph of distance moved over 5 min by CDCP1^GFP^ and CDCP1^GFP^-Y734F, -Y743F and -Y762F in response to 10D7 (5 µg/ml). Data are median and range from the 100 tracks with the highest velocity in each experimental group from three independent experiments. ***P<0.001.

As Src tyrosine phosphorylation is important in CDCP1-mediated signal transduction 12, 27, 30, we next examined this in antibody induced processing of CDCP1. Western blot analysis of lysates from HEY cells treated for up to 24 h with 10D7, indicated that 10D7 causes rapid phosphorylation of CDCP1-Y734 within 5 min with levels then gradually reducing as CDCP1 was degraded (Figure 5A). Interestingly, 10D7-induced phosphorylation of CDCP1 was accompanied by rapid transient activation of Src, indicated by auto-phosphorylation at Y416, over the same time course as p-CDCP1-Y734 (Figure 5A). To examine the role of the three known Src phosphorylation sites of CDCP1 30, 32 in 10D7-induced cellular processing, we employed live-cell confocal microscopy analysis of HEY cells stably expressing CDCP1^GFP^ or the Y734F, Y743F and Y762F mutants. Western blot analysis of cell fractions collected from these lines by cell surface biotinylation, confirmed that wildtype CDCP1^GFP^ and the mutants were expressed on the plasma membrane of HEY cells together with endogenously expressed CDCP1 (Figure 5B). We analysed live-cell images of the four cell lines to determine the distance moved (track length) by individual CDCP1^GFP^-positive puncta during mAb-induced internalization (Figure 5C, left). This showed that 10D7-induced internalization was significantly reduced in cells expressing Src phosphorylation site mutant CDCP1 compared to the wildtype protein (Figure 5C, right; median track length: wildtype (WT) 21.7 µm, Y734F 10.8 µm, Y743F 15.2 µm, Y762F 15.4 µm). Of note, none of the individual tyrosine mutations reduced track length to the background level induced by the control IgG, suggesting that Y734, Y743 and Y762 mediate, but are not essential for 10D7-induced internalization of CDCP1.

**Figure 6:**
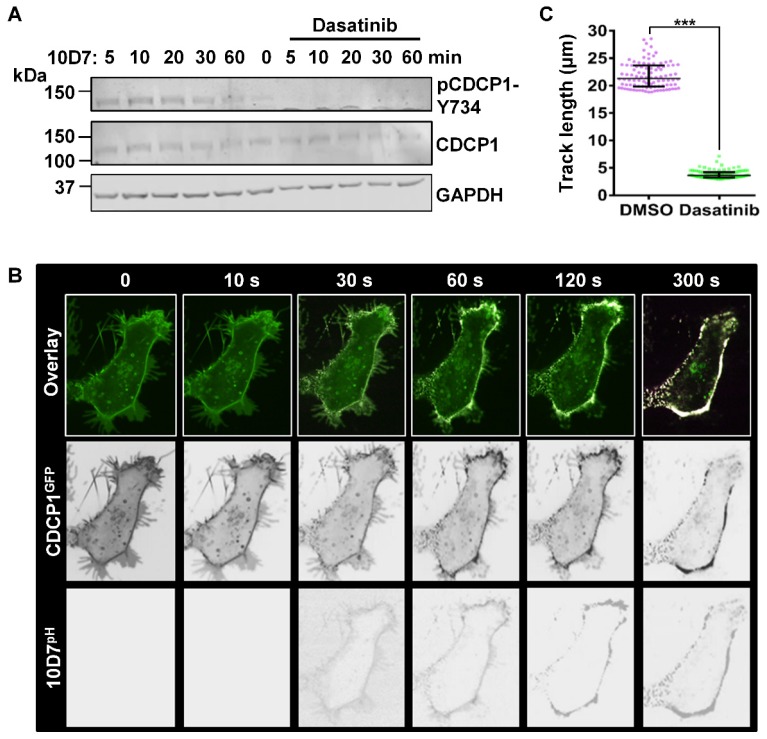
** The Src inhibitor dasatinib blocks 10D7-induced phosphorylation and internalization of CDCP1.** (**A**) HEY cells, treated for 2 h with dasatinib (200 nM), were incubated with 10D7 (5µg/ ml) for the indicated times. Lysates were examined by Western blot analysis for CDCP1 (1:2,000), pCDCP1-Y734 (1:2,000) and GAPDH (1:10,000). (**B**) Live-cell confocal microscopy images, acquired at the indicated time points after antibody treatment, of HEY-CDCP1^GFP^ cells pre-treated with dasatinib (200 nM), then incubated with 10D7^pH^. *Lower panels*, 10D7^pH^ signal. *Middle panels*, CDCP1^GFP^ signal. *Upper panels*, overlay of 10D7^pH^ and CDCP1^GFP^ signals. (**C**) Graph of distance moved over 5 min by CDCP1^GFP^ in response to 10D7 in the presence and absence of dasatinib. Data are median and range from the 100 tracks with the highest velocity in each experimental group from three independent experiments. ***P<0.001.

To further examine the role of tyrosine-phosphorylation in 10D7-induced CDCP1 internalization, we used the potent Src family kinase (SFK) inhibitor dasatinib 36. In addition to SFKs, dasatinib also potently inhibits BCR-ABL with lower potency against the kinases c-KIT, PDGFR, c-FMS and EPHA2 36. Pre-treatment with dasatinib completely blocked the generation of endogenous p-CDCP1-Y734 that is rapidly induced in response to 10D7 within 1 hour of treatment (Figure 6A). The effect on 10D7-induced CDCP1 internalization, examined by live-cell imaging, showed that in response to dasatinib both CDCP1^GFP^ and 10D7^pH^ remained largely at the plasma membrane (Figure 6B). Interestingly, although antibody and receptor were not internalized, overlapping signal from CDCP1^GFP^ and 10D7^pH^ in low pH cellular structures was apparent as dense white puncta located on the plasma membrane (Figure 6B). Consistent with the marked reduction in 10D7-induced internalization of CDCP1^GFP^, quantitative analysis indicated that dasatinib reduced CDCP1^GFP^ track length by ~80% (Figure 6c). Collectively these data demonstrate that 10D7-induced internalization of CDCP1 requires the action of a kinase, most likely Src, and that while phosphorylation of CDCP1 is evident, it is important but not essential for efficient 10D7-induced internalization of CDCP1.

### *In vivo* accumulation of 10D7 in EOC

**Figure 7:**
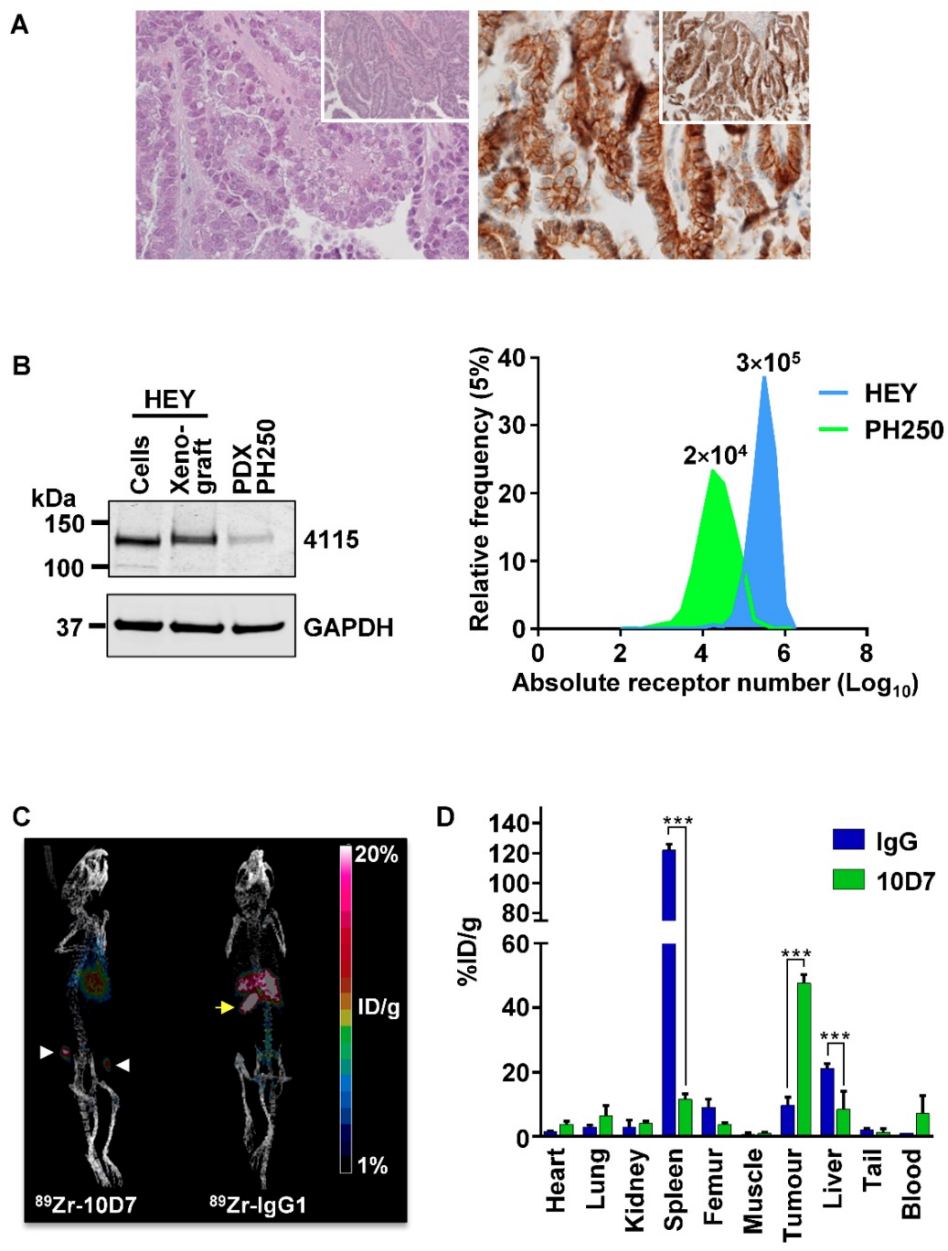
** PET-CT imaging of an EOC PDX.** (**A**) Clear cell EOC PDX PH250. *Left*, hematoxylin and eosin stained section highlighting clear cell features at 40X with 10X magnification (inset). *Right*, Anti-CDCP1 immunohistochemistry (antibody 4115) highlighting strong CDCP1 expression by malignant cells with accentuation of signal on the plasma membrane at 40X and 10X magnification (inset). (**B**) Comparison of CDCP1 expression by HEY cells and PDX PH250 cells. *Left*, Anti-CDCP1 (antibody 4115; 1:2,000) and GAPDH (1:10,000) Western blot analysis of lysates from HEY cells, a HEY cell xenograft, and a PH250 PDX tumor. *Right*, Cell surface CDCP1 receptor number determined by flow cytometry of single cell suspensions of HEY cells and PDX PH250 cells. Receptor numbers per cell are indicated above the flow cytometry peaks. (**C**) Representative PET images of mice carrying subcutaneous PH250 PDX tumors on both flanks. ^89^Zr-10D7 and ^89^Zr-IgG1κ were injected intravenously three weeks after tumor cell inoculation, and imaging performed 144 h later. White arrowhead, tumor nodules. Yellow arrow, ^89^Zr-IgG1κ signal accumulated in the spleen. (**D**) Quantitative bio-distribution analysis of ^89^Zr-10D7 and ^89^Zr-IgG1κ 144 h post injection (n = 4). 10D7 accumulates in tumors to a significantly higher degree than IgG1κ which accumulates in the spleen and liver. ***, P<0.001.

To investigate the potential for 10D7 to target CDCP1 expressing cells in EOC *in vivo*, we conducted a positron emission tomography (PET)-computed tomography (CT) imaging study. This was performed on mice bearing a previously described patient derived xenograft (PDX) designated PH250 that has confirmed clear cell EOC pathology (Figure 7A, left) 37. Immunohistochemical analysis indicated that CDCP1 is expressed predominantly on the surface of the malignant cells of this PDX (Figure 7A, right). Western blot analysis indicated that CDCP1 is expressed as the full-length 135 kDa form, which contains the region recognized by 10D7, and that its expression is lower than in HEY cells grown *in vitro* or as xenografts in mice (Figure 7B, left). Consistently, flow cytometry analysis established that cell surface CDCP1 receptor numbers are approximately 15 times higher on HEY cells (~300,000/cell) than cells isolated from PH250 xenografts (~20,000/cell) (Figure 7B, right). In this respect, PH250 xenografts were a more appropriate model than xenografts of HEY cells to first assess the sensitivity of a CDCP1-targeting to detect EOC *in vivo*.

PET imaging was first performed on mice with subcutaneous tumors in each flank, 3 weeks after injection of a slurry of PDX PH250 cells. 10D7 and IgG1κ were labelled with the positron-emitting radionuclide ^89^Zr, achieving chemical yields of 81% and 78%, respectively, with purity of >95%. Specific accumulation of ^89^Zr-10D7 but not ^89^Zr-IgG1κ (Figure 7C) in subcutaneous tumors was observed. *Ex vivo* bio-distribution analysis demonstrated percent injected dose per gram of tissue (%ID/g) values significantly higher in tumor for ^89^Zr-10D7 (47.7 ± 2.6 %ID/g) compared with ^89^Zr-IgG1κ (9.7 ± 2.5 %ID/g) (Figure 7D). Of note and consistent with the images in Figure 7C (right), ^89^Zr-IgG1κ showed significant accumulation in spleen (122.1 ± 3.9 %ID/g) and liver (21.2 ± 1.4 %ID/g) (Figure 7D). This contrasted with signals from five other normal organs, and the site of injection (tail) and blood, which were the same for ^89^Zr-labelled 10D7 and IgG (Figure 7D).

To better determine the potential of CDCP1 targeted contrast agents to detect EOC tumor burden in patients, PET imaging was also performed on mice carrying intraperitoneal tumors. As shown in Figure S1A, ^89^Zr-10D7 but not ^89^Zr-IgG1κ demonstrated specific accumulation in intraperitoneal tumors. *Ex vivo* bio-distribution analysis demonstrated %ID/g values significantly higher in tumor for ^89^Zr-10D7 (27.1 ± 16.0 %ID/g) compared with ^89^Zr-IgG1κ (5.2 ± 1.8 %ID/g; P = 0.017) (Figure S1B). This contrasted with signals from seven organs, blood and the injection site (tail), which were the same for ^89^Zr-labelled 10D7 and IgG (Figure S1A). The variability of the *ex vivo* biodistribution of 10D7 signal in tumors was due to difficulty in accurately weighing, *post mortem*, the large number of relatively small solitary tumor deposits which were generally < 3 mm in diameter.

These data indicate that 10D7-based agents effectively accumulate in CDCP1-expressing patient derived EOC tumors *in vivo*.

### Cytotoxin-conjugated 10D7 inhibits proliferation of CDCP1 expressing EOC cells *in vitro* and *in vivo*

**Figure 8:**
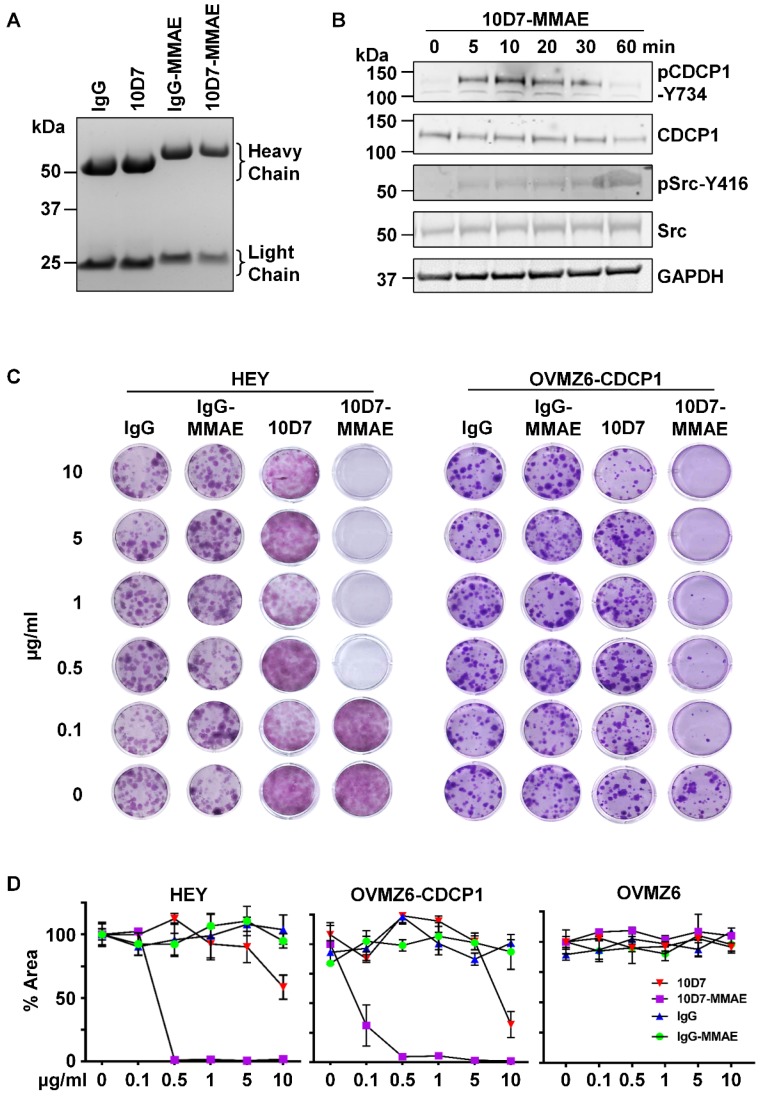
** 10D7-MMAE selectively inhibits colony formation of CDCP1 expressing but not non-expressing EOC cells.** (**A**) Commassie stained gel of IgG and 10D7, and purified products from reactions of IgG and 10D7 with MMAE. (**B**) HEY cells were treated with 10D7-MMAE (5 µg/ml) for the indicated times and lysates examined by Western blot analysis for CDCP1, p-CDCP1-Y734, Src, p-Src-Y416 and GAPDH. Antibody dilution was 1:2,000 except the anti-GAPDH antibody which was 1:10,000. (**C**) Representative images of crystal violet stained colonies formed from HEY and OVMZ6-CDCP1 cells after treatment with the indicated concentrations of IgG, IgG-MMAE, 10D7 or 10D7-MMAE. (**D**) Graph of crystal violet staining, as a percentage of area (% Area), of colonies formed by HEY, OVMZ6-CDCP1 and OVMZ6 cells after treatment with increasing concentrations of IgG, IgG-MMAE, 10D7 or 10D7-MMAE. Data represent means ± SEM from three independent experiments. ***, P<0.001.

We next examined the ability of 10D7 to target a cytotoxin for lysosomal release to inhibit colony formation of CDCP1 expressing EOC cells. 10D7 and control IgG1κ were labelled with the highly potent cytotoxin monomethyl auristatin E (MMAE) via a link incorporating a lysosomal protease cleavage site 38. Reducing SDS-PAGE analysis of the antibody-drug conjugates (ADCs) 10D7-MMAE and IgG-MMAE indicated that uniform MMAE labelling was achieved for each of the labelling reactions performed for the study, resulting in a molecular weight shift in the light and heavy chains of ~1.5 and ~4 kDa, respectively (Figure 8A). Consistent with these shifts, the average drug-antibody ratio (DAR) achieved for 10D7-MMAE from the six preparations of 10D7-MMAE was 4.5 to 4.7. Importantly, 10D7-MMAE was functionally active retaining the ability of the “naked” mAb to induce phosphorylation of CDCP1-Y734 and Src-Y416 within 1 hour of treatment (Figure 8B). To evaluate potency and selectivity, the anti-proliferative effects of 10D7-MMAE on HEY, OVMZ6 and OVMZ6-CDCP1 cells were compared with IgG-MMAE, 10D7 and IgG. While the control antibodies IgG, IgG-MMAE and 10D7 had little impact, colony formation of CDCP1 expressing HEY and OVMZ6-CDCP1 cells was sensitive to low concentrations of 10D7-MMAE (Figure 8C). Of note, OVMZ6 cells were unaffected by 10D7-MMAE even at the highest concentration, whereas the half maximum dose required to block colony formation of HEY and OVMZ6-CDCP1 cells was approximately 0.2 µg/ml and 0.07 µg/ml, respectively (Figure 8D). These data indicate that 10D7 can specifically deliver potent cytotoxic drugs to EOC cells via cell surface CDCP1 to inhibit proliferation.

### 10D7-MMAE reduces tumor burden and increases survival of a mouse model of EOC

**Figure 9:**
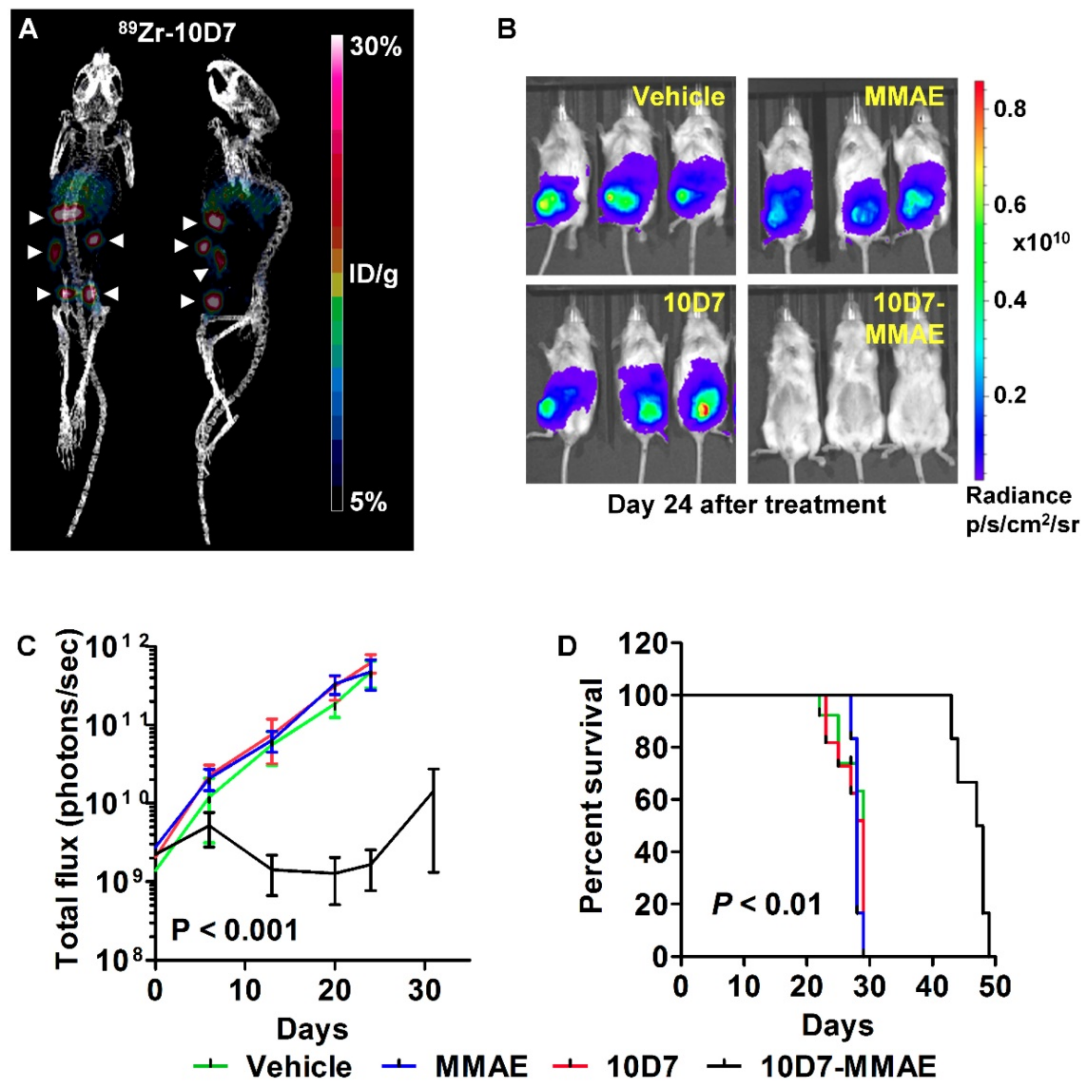
** 10D7-MMAE reduces tumor burden and increases survival of mice carrying intraperitoneal HEY cell xenografts.** (**A**) After 18 days of HEY cell growth (Day 0 in panel c), tumor burden was assessed in four randomly selected mice by PET-CT imaging using. ^89^Zr-10D7 was injected intravenously, and imaging performed 144 h later. *Left*, anterior-posterior view. *Right*, Lateral view. (**B**) Bioluminescence imaging. Immediately after PET-CT imaging the remaining mice were administered a single treatment of vehicle, MMAE, 10D7 or 10D7-MMAE. Bioluminsecence imaging was performed 7, 14, 21, 24 and 32 days later. Images from day 24 are shown. (**C**) Change in tumor burden quantified by bioluminescent imaging. (**D**) Kaplan-Meier survival analysis. Red arrow, day treatments administered.

We next assessed the anti-EOC effects of 10D7-MMAE *in vivo*. Mice were injected intraperitoneally with luciferase labelled HEY ovarian cancer cells. After 18 days of tumor growth, PET-CT imaging of four mice with ^89^Zr-10D7 consistently revealed distinct tumor nodules dispersed throughout the peritoneal cavity of mice including the pelvis, abdomen and diaphragm (Figure 9A). The remaining mice were randomized into groups of six then treated with a single dose of 10D7-MMAE or the controls 10D7, MMAE or vehicle. Bioluminescence imaging after another 24 days of growth, revealed significant tumor burden in mice treated with 10D7, MMAE and vehicle, but not in mice treated with 10D7-MMAE (Figure 9B). Quantification of the signal from regular bioluminescence imaging revealed that when control mice reached the ethical end-point and were euthanized, the tumor burden was approximately 100 times greater than in 10D7-MMAE treated mice (Figure 9C). Also of note, Kaplan-Meier analysis indicated that average survival of control mice was about 28 days, whereas it was 48 days for 10D7-MMAE treated mice (Figure 9D).

## Discussion

Analysis of pre-clinical models and patient samples indicate that CDCP1 promotes metastasis and chemotherapeutic resistance in a range of cancers 8-11, 14-16, 30, 39, 40, and this cell surface protein has been proposed as a target for antibody-based therapy for several malignancies 6, 9-11, 19-21, 24. Here we report novel insights on mechanisms by which two anti-CDCP1 mAbs, 10D7 and 41-2, induce rapid Src-mediated internalization and degradation of plasma membrane localized CDCP1 in EOC cells. Consistent with this ability to target a cancer promoting cell surface protein, radionuclide-conjugated mAb 10D7 was effective as a contrast agent for PET imaging of subcutaneous and intraperitoneal xenografts in mice, and this mAb was able to specifically deliver a cytotoxin to inhibit proliferation of CDCP1 expressing EOC cells *in vitro*, and growth of intraperitoneal xenograft tumors of EOC cells in mice. Considering its limited reported expression in normal tissues, including skin 41, colon 42 and prostate 43, these data support the potential utility of CDCP1 targeted agents to detect and treat CDCP1-expressing cancers such as EOC.

Consistent with the ability to inhibit migration and survival of cancer cells *in vitro* and xenograft growth *in vivo* 8-12, our data indicate that 10D7 and 41-2 bind extracellularly to CDCP1 within the region spanned by residues 30 to 358. These mAbs bind with high affinity causing rapid mAb/CDCP1 internalization followed by degradation. Demonstrating the versatility of 10D7 and 41-2 as experimental tools, both antibodies were able to recognise CDCP1 under the denaturing conditions of Western blot analysis as well as under the more native conditions of flow cytometry and live cell confocal microscopy. Interestingly, CDCP1 is internalized and degraded at the same rate in response to 10D7 as 41-2, despite the ~2.7 fold higher affinity of 10D7 for CDCP1 compared with 41-2 (0.44 nM versus 1.2 nM), and the ability of 10D7 to displace 41-2 from CDCP1 as determined by competition flow cytometry analysis. While it is not yet known whether the higher affinity of 10D7 is advantageous for its anti-cancer actions, we note that Herceptin, a therapeutic mAb clinically approved for breast cancer, has an affinity of 5 nM for its target HER2 44, and that optimal tumor targeting is in the 1-10 nM K_D_ range because higher affinity mAbs may be more rapidly degraded thereby limiting tumor penetration 45, 46.

Live-cell imaging indicated that 10D7 causes rapid clustering of CDCP1 at the cell surface and colocalization of the mAb and receptor, including within low pH vesicles, followed by degradation of CDCP1/mAb complexes. It will be important to accurately define the various vesicles to which CDCP1 and 10D7 traffic during internalization and degradation, potentially by co-staining cells with fluorescent markers that highlight the relevant cellular structures. A previous study reported on the mode of action of another anti-CDCP1 mAb, RG7287, which also has impressive anti-cancer properties including the ability to substantially slow growth of mouse xenografts 21. Similar to 10D7 and 41-2, RG7287 induces transient Src-mediated phosphorylation of CDCP1 followed by receptor internalization and degradation. The timeframe for these processes is more similar to 41-2-induced effects than 10D7, possibly reflecting the affinity of RG7287 for CDCP1 of 1.2 nM 21 the same as 41-2. Interestingly, while 10D7-induced CDCP1 degradation is via proteasomal and lysosomal mechanisms, RG7287-mediated degradation was almost exclusively proteasomal 21. Consistent with our observation of 10D7-induced clustering of CDCP1, RG7287-induced translocation of CDCP1 to detergent insoluble plasma membrane fractions which required antibody bivalency suggested CDCP1 dimerization or clustering 21. These findings indicate that mAb-ligated CDCP1 could be organised into specific membrane compartments to associate with other membrane or intracellular proteins as cargo for trafficking in a mAb-dependent manner for proteasomal and/or lysosomal processing.

Our observation that 10D7-induced phosphorylation of CDCP1 is accompanied by rapid transient activation of Src at Y416, is consistent with previous reports showing that CDCP1-associated Src is auto-phosphorylated at this site. It occurs in settings where cells become detached 28, and during CDCP1 cleavage 31 and elevated expression 32. The mechanisms by which these diverse signals drive Src auto-activation remain to be explored. However, to address the potential importance of Src self-activation and its phosphorylation of CDCP1 to antibody-induced cellular processing of CDCP1, we employed the SFK and BCR-ABL inhibitor dasatinib. The results were impressive with dasatinib completely blocking 10D7-induced internalization and degradation of CDCP1. Because of the dual potency of this inhibitor for SFKs and BCR-ABL, it will be important to employ more selective SFK inhibitors in these assays. It will also be important to directly address whether it is SFK-mediated phosphorylation of CDCP1, and not another substrate, that contributes to such rapid 10D7-induced internalization and degradation of CDCP1.

In addition to the involvement of Src in these processes, dephosphorylation is also likely required as a recent study indicated that the oncogenic tyrosine phosphatase SHP2 regulates phosphorylation and antibody-induced internalization of CDCP1 47. Also, it is important to note that the effect of CDCP1 cleavage on antibody induced internalization and degradation is not yet known. Antibodies 10D7 and 41-2 bind the amino terminal region of CDCP1 that is upstream of the protease cleavage sites (R368 and K369). For clinical applications it will be important to determine whether levels of intact CDCP1 present on the cell surface are sufficient for effective imaging and/or treatment by agents that target the amino terminal of this receptor.

To explore whether the ability of anti-CDCP1 mAbs to induce rapid internalization and degradation of CDCP1 can be further exploited against cancer cells, we examined the effectiveness of conjugated forms of 10D7 as agents for PET imaging of cancer *in vivo*, and inhibition of cell proliferation *in vitro* and tumor growth *in vivo*. Our data indicate that ^89^Zr-conjugated 10D7 is effective for PET imaging of tumors that display varying levels of CDCP1 and at both subcutaneous and intraperitoneal locations. Of note, ^89^Zr-10D7 was able to detect small intraperitoneal PDX PH250 tumor nodules of < 3 mm in diameter. Because PH250 cells expresses CDCP1 at levels about 15 times lower than the EOC cell line HEY, these data suggest that PET imaging approaches could be effective at detecting small solitary, low CDCP1 expressing tumor deposits in EOC patients potentially augmenting or replacing ultrasound for cases with low volume disease at diagnosis or recurrence.

Based on the growing literature reporting the functional roles of CDCP1 in resistance to therapies in various cancer types 11, 16, 17, 48, 10D7-based imaging may also be useful in measuring responsiveness to targeted therapies. CDCP1 was reported to promote resistance to trazatuzumab in HER2 positive breast cancer 16, 48, nilotinib in chronic myeloid leukaemia 17 and carboplatin resistance in clear cell EOC 11. Furthermore, increased CDCP1 expression has been linked to metastasis in cancers that also express moderate levels of CDCP1 in normal epithelium, including colon and lung cancer 23, 26, 49. In particular in colon cancer, high CDCP1 and CD110 expression has been reported to define a population of migrating cancer stem cells *in vivo* 50. In these cancers targeting CDCP1 with an ADC may not be appropriate due to unintended effects on normal epithelium, but labelled anti-CDCP1 antibodies may be of greater use as imaging agents to detect metastases, highlighting the potential of this antibody as a versatile molecular tool for cancer.

Also of note, an ADC incorporating 10D7 and the highly toxic agent MMAE had specific and potent anti-growth activity against CDCP1-expressing EOC cells *in vitro* with no effect on non-expressing cells. Further highlighting specificity, an MMAE-labelled isotype matched IgG had no impact on growth of two high CDCP1 expressing cell lines *in vitro*. Importantly, 10D7-MMAE also displayed potent anti-EOC effects *in vivo*, slowing progression of HEY cell xenografts grown intraperitoneally in mice, and significantly prolonging mouse survival. In EOC, increased CDCP1 expression has been reported in significant proportions of tumors compared with normal fallopian tube or ovary, including in 77% of high grade serous ovarian cancer cases 10 and 90% of clear cell ovarian cancer cases 11, indicating that a large proportion of EOC cases may benefit from CDCP1 targeted agents for imaging or treatment. Clear cell EOC in particular is characterized by relative chemotherapy resistance and a high recurrence rate, making this histologic subtype challenging to treat clinically. It is important to note that in EOC patient tumors CDCP1 expression levels have varied between weak, moderate and strong and the proportion of CDCP1 expressing malignant cells has not been reported 10, 11. An important issue for the effectiveness of CDCP1-trageted agents for imaging and treatment of EOC and other cancers will be the proportion of malignant cells that express this receptor on the cell surface. This is particularly important for patients typically receiving ADC therapies who generally will have been heavily pre-treated with other agents. These treatments can reduce expression of the targeted antigen, thereby reducing the efficacy of the cancer targeting agent. In addition, as the ECD of CDCP1 is known to be proteolytically processed 31, 42 it will be important to understand how proteolysis impacts the effectiveness of CDCP1-targeted agents for cancer detection and treatment. Accordingly, further work is required to determine the CDCP1 expression levels and cellular processing that are effective PET imaging and treatment. Nevertheless, extrapolating from our data, it is possible that CDCP1-targeted agents will display efficacy for detection and treatment of EOC and other CDCP1 expressing cancers.

## Methods

### Antibodies and reagents

Antibodies 10D7 and 41-2 were described previously 15, 49. Rabbit anti-CDCP1 (#4115), rabbit anti-p-CDCP1-Y734 (#9050), mouse anti-Src (#2110) and rabbit anti-p-Src-Y416 family (#2101) antibodies were from Cell Signaling Technology (Gold Coast, Australia). Mouse anti-GAPDH antibody was from Merck (Kilsyth, Australia). PE-conjugated anti-CDCP1 antibody CD318^-PE^ and APC-conjugated anti-mouse secondary antibody were from BioLegend (Karrinyup, WA, Australia). Isotype control IgG1κ was from Sigma-Aldrich (Castle Hill, Australia). Recombinant CDCP1-ECD, spanning residue 30 to 665, was described previously 51. Maleimidocaproyl-valine-citrulline-p-aminobenzoyloxycarbonyl-MMAE (MC-VC-PAB-MMAE) was from Levena (San Diego, CA).

### Expression constructs, cell culture, transfections and cell treatments

Y734F, Y743F and Y762F mutants were introduced into a described CDCP1 expression construct 31 by site directed mutagenesis. CDCP1^GFP^ construct was sub-cloned from a described construct 31 into vector pEGFP-N1 (Clontech, Mountain View, CA). Culture media and reagents were from Thermo Fisher Scientific (Scoresby, Australia) and plasticware from Corning (Mulgrave, Australia). HEY (ATCC, Manassas, VA) and OVMZ6 9 EOC cells were cultured in RPMI and DMEM media, respectively, containing 10% (v/v) FCS (HyClone, In Vitro Technologies, Eight Mile Plains, Australia), penicillin (100 units per ml) and streptomycin (100 units per ml), at 37°C. OVMZ6 cell media contained 2 mM sodium pyruvate and 2 mM L-glutamine. Lipofectamine 2000 was used for transfections 9, 32 to generate OVMZ6-CDCP1 cells and HEY cells stably expressing wildtype, Y734F, Y743F or Y762F CDCP1^GFP^. For microscopy 4-chamber 35 mm glass-bottom dishes (Cellvis, Mountain View, CA) were coated with poly-L-lysine (Sigma-Aldrich) then dried before cell plating. Cell treatments were: dasatinib (200 nM; Sigma-Aldrich) for 2 h before antibody as described 36; proteasome inhibitor MG132 (20 µM; Sigma-Aldrich) and lysosome acidification inhibitor chloroquine (50 µM; Sigma-Aldrich) were added 16 h before antibody.

### Flow cytometry

Adherent cells lifted non-enzymatically were fixed (4% paraformaldehyde; 30 minutes) then incubated with 10D7 or 41-2 (5µg/ ml) in PBS/1% BSA (30 minutes; 4°C). PBS washed cells were stained with an APC-conjugated anti-mouse secondary antibody in PBS/1% BSA (30 min; 4°C). For assays assessing impact of antibodies on cell surface CDCP1, adherent HEY and OVMZ6-CDCP1 cells were untreated or treated with 10D7, 41-2 or IgG_1_κ (5µg/ml; 30 min; 37°C) in complete medium then lifted non-enzymatically before staining (30 min, 4°C) with antibody CD318^-PE^. After washes, 20,000 events were analyzed on a BD Accuri C6 flow cytometer (BD Bioscience, North Ryde, NSW, Australia) with data displayed as mean fluorescence intensity (MFI) calculated by subtracting the value from staining only with secondary antibody.

Assays quantifying the number of cell surface CDCP1 receptors were performed by flow cytometry as described previously 52 using the anti-CDCP1 antibody CD318^-PE^ and QuantiBRITE PE beads (BD Biosciences). HEY cells and cells isolated from a freshly harvested PH250 xenograft (1×10^5^) were incubated at room temperature for 1 h with a dilution series of CD318^-PE^ (0.25, 0.5 and 1 µM). Flow cytometry analysis identified a saturating concentration of CD318^-PE^ molecules and a corresponding MFI value which was used to extrapolate the number of CDCP1 receptors per cell from a standard curve of the log_10_ values for the number of PE molecules per QuantiBRITE PE bead against the log_10_ values for MFI values.

Assays to assess the competition of antibodies 10D7, 41-2 and CD318^-PE^ for binding sites on CDCP1 were performed by flow cytometry using two approaches. The first employed 10D7 labelled with the fluorescent dye QDot^625^ using a SiteClick antibody labeling kit (Thermo Fisher Scientific). HEY cells (1×10^5^) were incubated with either 10D7-QDot^625^ for 1 h, unlabelled 41-2 for 1 h then 10D7-QDot^625^ for 1 h, or concurrently with 10D7-QDot^625^ and 41-2 for 1 h. In the second approach, HEY cells (1×10^5^) were incubated with CD318^-PE^ for 1 h, unlabelled 10D7 or 41-2 for 1 h then CD318^-PE^ for 1 h, or concurrently with 10D7 or 41-2 and CD318^-PE^ for 1 h. All antibodies were at the saturating concentration of 2 µM. Flow cytometry detected fluorescence of 10D7-QDot^625^ or CD318^-PE^.

### Antibody affinity

Surface plasmon resonance was performed using a Biacore T200 (GE Healthcare, Parramatta, Australia) as described 53 with antibodies immobilized via Protein G (Sigma-Aldrich) on a CM5 chip (GE Healthcare). Binding kinetics to immobilized mAbs was of serial dilutions of CDCP1-ECD (50 to 1.56 nM; 30 µl/min) with 180 s association and 600 s dissociation time at 25°C. Data were processed using BIAevaluation software with readings double-referenced by subtraction of a “buffer only” control against the reference-subtracted sensorgrams.

### Antibody internalization

HEY and OVMZ6-CDCP1 cells were adhered overnight in 96-well black-walled, clear-bottom plates (10,000 cells/well). Using amine chemistry, antibodies were conjugated with the hydrophilic, bright pH sensor dye, pHAb (Promega, Alexandria, Australia), that fluoresces at acidic pH within endosomes and lysosomes 34. Adherent cells were incubated at 37°C with pHAb-conjugated antibodies (5µg/mL) with signal acquired at defined time points using a fluorescent plate reader (excitation 532 nm; emission 560 nm).

### Live-cell microscopy

Live-cell spinning-disk confocal imaging was performed as described 54 on an environmentally controlled Nikon TI inverted microscope (Nikon, New York) equipped with a Borealis-modified Yokogawa CSU-X1 confocal head (Spectral Applied Research, Ontario Canada) and a Clara cooled interline charge-coupled device (CCD) camera (Andor Technology, Belfast, United Kingdom). Dynamics of fluorescent proteins were imaged at 37°C using a 60× 1.49 NA objective (Nikon) with imaging of CDCP1^GFP^ internalization induced by 10D7^pH^ performed at ~1 frame/sec. Image analysis and quantification was performed using Imaris 7.1 software (Bitplane, Zurich, Switzerland). Briefly, the Spots creation wizard automatically subtracted background and detected CDCP1^GFP^ puncta which were filtered for analysis based on threshold values above 20 for 'quality' defined as the intensity at the centre of the puncta, Gaussian filtered by ¾ of the spot radius 55. Puncta were tracked over sequential frames using the Autoregressive motion particle-tracking algorithm. A maximum search distance of 0.9 µm was defined to prevent false track connections to nearby spots. A gap-closing algorithm linked track segment ends to track segment starts to recover tracks that were interrupted by temporary particle disappearance. Maximum permissible gap length was set to 3 frames. Three independent assays were performed for each experiment. Quantification of track length during the period 0-5 min was determined from the 100 tracks with the highest velocity in each experimental group. The software generates images overlaying color-coded tracks onto cells, with colors corresponding to the visible spectrum (violet, tracks that moved the shortest distance; red, tracks that moved the greatest distance). The intensity of CDCP1^GFP^ and 10D7^pH^ fluorescence signal was quantified using ImageJ software employing a previously described method 56. Images from the indicated time frames were split into RBG colour channels and the GFP channel was converted to a binary mask to delimit CDCP1^GFP^ and 10D7^pH^ puncta and generate selections of individual GFP-positive and 10D7^pH^-positive 'particles' as regions of interest (ROI). The mean gray value (the sum of the gray values of all the pixels in the selection divided by the number of pixels) of 10D7^pH^-positive puncta overlapping each CDCP1^GFP^-positive particle per ROI was determined and graphed.

### Cell surface biotinylation and Western blot analysis

Cells were biotinylated (4°C, 1 h) with cell-impermeant EZ-link NHS-SS Biotin (1.22 mg/ml; Thermo Fisher Scientific) then PBS washed and lysed in buffer containing 20 mM HEPES, 150 mM NaCl, 1 mM EDTA, 1% Triton X-100, 1× Complete protease inhibitor cocktail (PIC) and phosphatase inhibitors (2 mM Na_3_VO_4_, 10 mM NaF). After centrifugation the supernatant was incubated (15 minutes, 4°C) with ImmunoPure immobilized streptavidin beads (Thermo Fisher Scientific). After pelleting, the supernatant containing intracellular proteins was transferred to a separate tube, and the beads washed in lysis buffer containing PIC and phosphatase inhibitors. Lysates and proteins separated by cell surface biotinylation (40 µg/lane) were examined by Western blot analysis as described 11 with antibody 10D7 and 41-2 used at 1 µg/ml, anti-GAPDH antibody at a dilution of 1:10,000 and all other antibodies at 1:2,000 dilution. After overnight incubation in primary antibodies membranes were washed with Tris-buffered saline containing 0.1% Tween 20, and then incubated with IRDye 680- or 800-conjugated secondary mouse or rabbit IgG as appropriate. Images of membranes and densitometric quantification of signals were generated using the Odyssey system and software (LI-COR Biosciences, Millennium Science).

### PET-CT imaging of an intraperitoneal clear cell EOC PDX

Mouse experiments were approved by the University of Queensland Animal Ethics Committee. For PET/CT imaging female NOD.Cg-*Prkdc^scid^ Il2rg^tm1Wjl^*/SzJ (NSG) mice (6-8 weeks; 4 per group; Jackson Laboratory, Bar Harbor, ME) were injected subcutaneously or intraperitoneally with cells dissociated from a previously described clear cell EOC PDX designated PH250 37 (0.2 g/mouse of pelleted cell slurry). CDCP1 expression by PDX PH250 was assessed by immunohistochemical and Western blot analysis as described 11. The number of CDCP1 receptors present on the cell surface was determined by flow cytometry as described above. 10D7 and IgG1κ control were labelled with the positron-emitting radionuclide ^89^Zr as described 57, and yield and purity determined by radio-TLC and -HPLC (Agilent, Mulgrave, Australia). Imaging commenced 3 weeks after injection of cancer cells which was sufficient time for tumors to establish at subcutaneous and intraperitoneal sites, and before the accumulation of large, dense ascites from intraperitoneal tumors. Imaging was performed on isoflurane anaesthetised mice injected via the lateral tail vein with 3-5 MBq of ^89^Zr-10D7 or ^89^Zr-IgG1κ, and was performed after 1, 24, 48, 72 and 144 h using an Inveon PET/CT (Siemens, Munich, Germany). PET acquisition (30 minutes; static emission) was performed, and images were reconstructed using an ordered-subset expectation maximization (OSEM2D) algorithm, with CT attenuation correction. The CT scan parameters were 80 kV, 500 µA, 230 ms exposure time, 360^o^ rotation with 180 rotation steps, binning factor of 4, low magnification position, producing an effective pixel size of 106 µm, with CT images reconstructed using the Feldkamp algorithm. All PET and CT images were reconstructed using Inveon Acquisition Workplace software (Siemens). PET activity per voxel was converted to bq/g using a conversion factor obtained by scanning a cylindrical phantom filled with a known activity of ^89^Zr to account for PET scanner efficiency. Activity concentrations within tissue ROIs were expressed as percentage of the decay-corrected injected activity per gram of tissue (%ID/g; SUV) using Inveon Research Workplace software (Siemens). *Ex vivo* bio-distribution was assessed after the final imaging time point. Harvested tumor and organs, cleaned of blood, were weighed and radioactivity quantified using a Wizard 2480 gamma counter (Perkin Elmer) and presented as %ID/g of tumor or tissue (after decay and detector efficiency corrections).

### Antibody-drug conjugation

MMAE-conjugated 10D7 and IgG1κ were prepared as described 58. Maleimidocaproyl-valine-citrulline-p-aminobenzoyloxycarbonyl-MMAE (MC-VC-PAB-MMAE) was from Levena (San Diego, CA). First, antibody inter-chain disulfides were partially reduced by incubating 10D7 (5 mg/ml) with DTT (10 mM) for 15 min at 37°C to generate free thiols. After buffer exchange to remove unreacted DTT, the partially reduced antibody was reacted with a 10 fold molar excess of maleimide activated MC-VC-PAB-MMAE to generate the crude 10D7-MMAE product. Reaction impurities were removed by ultrafiltration. DARs were determined by reverse phase LC/MS analysis of the reaction mixtures containing separated light and heavy chains as reported 59. Chromatograhic separation for DAR analysis was through a PLRP-S 1000 Å, 5 µm, 50 × 2.1 mm column (catalogue number PL1912-1502, Agilent) on a Shimadzu Nexera 1D UHPLC system (Shimadzu, Rydalmere, Australia) coupled to an ABSCIEX Triple TOF spectrometer (AB SCIEX Framingham, MA). The number of drugs per light and heavy IgG chain was determined by mass spectroscopy analysis of each fraction. The mean DAR was then calculated by addition of the relative proportion of the area under the curve of each fraction 60, 61. In total six ADC reactions were performed generating products with average DARs of 4.5 to 4.7, including a DAR of 4.5 for the preparation used for the *in vivo* study.

### Colony formation analysis

Colony-formation assays were performed as described 62 on cells (100,000/ml) treated with the indicated concentrations of 10D7, 10D7-MMAE, IgG or IgG-MMAE overnight before being lifted and replated in 24-well plates (200 cells/well). After a defined growth period media was removed and cells gently PBS washed before staining with 0.1% crystal violet (Sigma-Aldrich) in 2% ethanol. After 20 minutes and stained cells were scanned (700 nm) on a LiCOR System (Odyssey V3.0 software) with images analyzed using the ColonyArea ImageJ plugin to quantify colony area and intensity 63.

### Mouse assays assessing ADC effect on tumor burden and survival

Female NSG mice (6-8 weeks; 28 mice) were injected intraperitoneally with luciferase labelled HEY cells (5× 10^5^). After 18 days, tumor burden was assessed in four mice by PET-CT imaging using ^89^Zr-10D7 as described above. Once tumor burden was confirmed the remaining mice were randomized into groups of six and administered a single intravenous treatment of 10D7-MMAE (5 mg/kg), 10D7 (5 mg/kg), MMAE (0.17 mg/kg; equivalent to a four molar excess of the 10D7-MMAE dose) or vehicle. Tumor growth was monitored by bioluminescent imaging as described previously 10, 64. Mice were culled when tumor burden or animal discomfort exceeded ethically approved limits, with survival examined by Kaplan-Meier analysis as described 65.

### Statistical Analysis

*In vitro* assays were performed in triplicate on three independent occasions. Analyses used GraphPad Prism (GraphPad, La Jolla, CA) with data displayed as mean and standard error of the mean (SEM) or for non-parametric data, median and range (10-90 percentiles). Statistical significance was assessed by One-way ANOVA or Student's t-test for parametric data, and for non-parametric data the Mann-Whitney test (t-test) or Kruskal-Wallis ANOVA, with P-value <0.05 considered significant.

## Supplementary Material

Supplementary figure.

## Abbreviations

CDCP1: CUB domain containing protein 1
mAb: monoclonal antibody
MMAE: monomethyl auristatin E
ADC: antibody drug conjugate
EOC: epithelial ovarian cancer
SFK: Src family kinase
