# Supplementary material for: Anti-CDCP1 immuno-conjugates for detection and inhibition of ovarian cancer
Source: Theranostics. 2020 Jan 12;10(5):2095–114. doi: 10.7150/thno.30736 (PMC7019151; doi:10.7150/thno.30736)
Supplement: Supplementary file 1 — Supplementary figure. [file thnov10p2095s1.pdf]

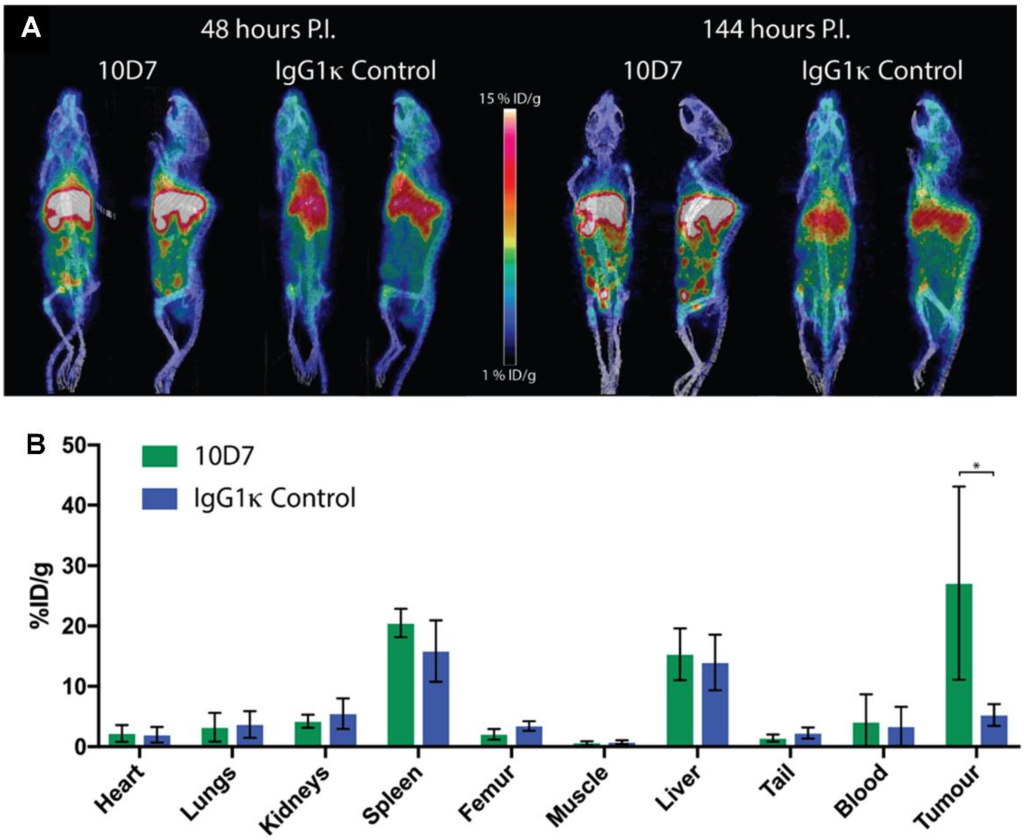

Figure S1. PET-CT imaging of an intraperitoneal EOC PDX. (**A**) PET images of mice three weeks after intraperitoneal injection of a cell slurry of the clear cell EOC PDX PH250. Imaging was performed at 48 and 144 h post injection (P.I.) of ^{89}Zr-10D7 and ^{89}Zr-IgG1κ. 10D7 accumulates in tumors whereas IgG1κ does not. (**B**) Quantitative bio-distribution analysis of ^{89}Zr-10D7 and ^{89}Zr-IgG1κ 144 h post injection (n = 4). 10D7 accumulates in tumors to a significantly higher degree than IgG1κ (P = 0.017).
